# Impact of the Dog–Human Bond on Canine Social Evaluation: Attachment Predicts Preference toward Prosocial Actors

**DOI:** 10.3390/ani13152480

**Published:** 2023-08-01

**Authors:** Emily M. Richards, Zachary A. Silver, Laurie R. Santos

**Affiliations:** Department of Psychology, Yale University, New Haven, CT 06520, USA; zsilver@oxy.edu (Z.A.S.);

**Keywords:** social evaluation, attachment, domestication, *Canis familiaris*

## Abstract

**Simple Summary:**

The human species naturally judges whether other agents are nice or mean from a young age. Recent research has suggested that such social judgments are influenced by the way humans form attachment bonds with others. Given dogs’ rich evolutionary history alongside humans, researchers have become interested in whether dogs make similar evaluations of human social interactions, for instance, by distinguishing between someone who is helpful or unhelpful. However, this concept, to date, has shown mixed results. In the present study, we explore whether dogs’ attachment bonds impact their ability to form these judgments. Specifically, the present study sought to investigate whether dogs’ attachment bonds to their owners could predict the extent to which they successfully evaluated unfamiliar humans who interacted with their owners. We found that dogs with stronger attachment bonds to their owners were more likely to prefer people who helped their owners but were no more likely to avoid people who refused to help their owners. These results suggest that, as in humans, a dog’s attachment may impact the way that they evaluate potential social partners.

**Abstract:**

Scholars have argued that social evaluation, the capacity to evaluate different potential social partners, is an important capacity not just for humans but for all cooperative species. Recent work has explored whether domesticated dogs share a human-like ability to evaluate others based on prosocial and antisocial actions toward third parties. To date, this work has shown mixed results, suggesting that individual differences may play a role in dogs’ capacity to evaluate others. In the present study, we test whether attachment—an individual difference that affects human social evaluation performance—can explain the mixed pattern of social evaluation results observed in dogs. We first tested dogs on a social evaluation task in which an experimenter either helped or refused to help the dog’s owner open a container. We then assessed dogs’ attachment strength using a subset of the C-BARQ. We found that attachment was a statistically significant predictor of dogs’ preference toward the prosocial actor but was not a predictor in antisocial or control conditions. This finding provides early evidence that attachment may drive positivity biases in dogs and that attachment might explain mixed results within canine social evaluation literature.

## 1. Introduction

The ability to recognize and evaluate the actions of others is an important skill for any cooperative species. Many scholars have argued that the capacity to distinguish prosocial others—individuals who are likely to be helpful or cooperative in the future—from antisocial others—individuals who may be selfish and uncooperative—could be beneficial for the survival of social animals, e.g., [1,2]. Much research has shown that social evaluation is a critical skill in the human species and is one that develops very early in life. Human infants as young as 3 months in age prefer novel agents that behave prosocially (e.g., helping another agent to achieve this goal) relative to agents that behave neutrally and choose to avoid novel agents that behave antisocially (e.g., preventing an agent from achieving his goal) relative to neutral agents, see [3,4,5,6,7]. In one classic study [3], infants watched as a puppet tried and failed to make it up a steep hill. Infants were then introduced to two new puppets: one who acted prosocially, helping the first character up the hill, and a second who acted antisocially, hindering the first character by pushing him down the hill. When given a choice between the two puppets, infants reliably preferred to interact with the prosocial over the antisocial puppet. Results like these suggest that some capacity to evaluate the actions of agents is present within the first few months of human life.

The early emergence of social evaluation in the human species has prompted comparative researchers to explore whether similar capacities exist in other non-human species or whether such abilities are instead unique to humans. To test this question, researchers first explored whether non-human primates possessed the ability to socially evaluate novel agents. Krupenye and Hare [8] presented bonobos with a task similar to the ones used to test human infants and found that, in contrast to the performance of human infants, bonobos preferred antisocial humans. However, not all primates appear to show this antisocial preference. For example, Kawai et al. [9] found that marmoset monkeys avoided third parties who did not reciprocate during a social exchange. Similarly, Anderson and colleagues found that tufted capuchin monkeys tended to avoid antisocial humans who explicitly refused to help a third party [10] or failed to reciprocate goods with another actor [11]. Other studies have observed that non-human primates appear to make some social judgments when socially eavesdropping on prosocial or antisocial actors, but only sometimes and often with varying effects [12]. For example, Herrmann et al. [13] directly compared the social evaluative capacities of human children and non-human great apes (chimpanzees, bonobos, and orangutans) and found that human children and orangutans preferred a prosocial human actor when they themselves were the direct recipient of the actor’s actions, while chimpanzees and bonobos did not exhibit any preference. Interestingly, Russell et al. [14] found nearly the opposite pattern of results; they observed that chimpanzees tended to prefer a prosocial actor—who gave food to a begging experimenter—compared to an antisocial actor but found that orangutans, gorillas, and bonobos exhibited no preference [14]. Taken together, studies on primate social evaluation to date show a rather mixed pattern of results, suggesting that non-human primates might possess a more limited capacity for social evaluation than developing human infants and children.

Other researchers have begun to explore whether non-human animals share human-like social evaluation capacities by focusing on a different group of non-human subjects, ones that have more experience interacting with human agents: domesticated pet dogs (*Canis familiaris*). Many researchers have argued that dogs might be an especially good species to test for social evaluation, given the close domestication history that dogs have shared with humans [1]. However, such canine social evaluation studies have also yielded mixed results to date: see the review in Silver et al. [15]. Some studies have found that dogs prefer prosocial over antisocial humans [15,16,17,18], whereas many other studies have found that dogs show no significant preferences when choosing between prosocial and antisocial individuals [19,20,21,22,23,24].

Researchers have now begun to investigate why dogs show such mixed performance on social evaluation tasks. Some researchers have begun testing whether specific methodological factors can explain the extent to which dogs are able to socially evaluate agents. Freidin et al. [22], for example, found that dogs are more likely to distinguish between prosocial and antisocial agents when they are given more explicit body language and verbal reactions to help them distinguish between the behavior of different agents. Similarly, Carballo et al. [17] found that dogs were successfully able to distinguish prosocial and antisocial human actors when those actors were of different genders but not when the two actors were of the same gender. In another example, Chijiiwa and colleagues [25] investigated whether dogs might show a more human-like pattern of social evaluation when they have a close relationship with the third-party individual who is being helped or hurt. To test this question, Chijiiwa and colleagues presented dogs with people who directed prosocial and antisocial actions not toward strangers, as in most studies, but instead toward the dogs’ owners. Interestingly, Chijiiwa and colleagues found that dogs did not prefer individuals who helped their owners over neutral individuals who did not interact with their owner and instead showed a bias against antisocial individuals that no previous studies had found [25]. Other studies have taken a different approach to understanding dogs’ mixed performance in social evaluation tasks, examining whether individual differences in dogs’ backgrounds or training could explain the pattern of effects observed in social evaluation studies. Silver et al. [15], for example, found that trained agility dogs showed a human-like pattern of preferring prosocial to antisocial experimenters, whereas untrained pet dogs showed no preference.

The present study aims to explore whether another stable individual difference can explain the mixed pattern of results observed in canine social evaluation studies. Specifically, this study explores whether the way that dogs form relationships with humans can serve as a factor in determining whether dogs show preferences for prosocial or antisocial actors. Researchers in human psychology have found that attachment bonds—the emotional bond from one individual to another—emerge early in human development and form during early interactions between infants and their primary caregivers [26]. The nature of a young child’s attachment to their primary caregiver has wide-reaching impacts on their development. For instance, the nature of this bond has a strong impact on the child’s feelings of safety and security in the presence and absence of their primary caregiver as well as on their willingness to engage with novel stimuli and social partners [26].

Furthermore, these early attachment experiences form a relatively stable foundation for how we approach, develop, and maintain close relationships even through adulthood [27,28]. Research in adult humans has shown that a person’s attachment style—one of several predictable patterns of attachment—can impact their preference for cognitive closure and how likely they are to use new information during social evaluations [29]. Similarly, research has shown that attachment style can predict a person’s level of social curiosity, e.g., [29,30], and their sensitivity to social expressions [31]. Adults with different attachment styles also show differences in neural activation during social appraisals [31] and in their level of attentional control during non-social tasks [32]. Importantly for the purposes of the present experiment, new work has also shown that a person’s early attachment affects their responses to prosocial versus antisocial behaviors during social evaluation paradigms, e.g., [33,34,35]. These findings suggest that there may be an important connection between attachment and social evaluation in humans.

Given that attachment appears to be a meaningful individual difference in human social preferences, this study aimed to explore whether similar individual differences in dog attachment could explain the mixed performance that dogs exhibit in standard social evaluation studies. While much work has examined the nature of human attachment (including that of human-to-dog attachment, see [36,37]), less work has tested the nature of dog-to-human attachment and non-human–animal-to-human attachment more broadly. However, a growing body of work has hinted that dogs may exhibit stable individual differences in the attachments they set up with others, e.g., [38,39,40,41,42,43,44,45,46,47,48,49], with the characteristics of dogs’ patterns of attachment to their owners closely resembling those of human infants to their primary caregivers [45,46]. Additionally, emerging evidence has suggested that dogs’ relationships with their owners seem to impact their behavior in cognitive tests. For example, the nature of this relationship appears to impact dogs’ performances during problem-solving tasks [48,50] and their heart rate responses during threatening situations [51]. Taken together, these results provide evidence that, like humans, dogs’ attachment bond to their owners appears to be a stable individual difference impacting their performance in cognitive tasks [48] and their willingness to engage with novel social partners [52].

The goal of the present study was to test whether a dog’s attachment also affects the dog’s success in social evaluations. To test this question, dogs were presented with the social evaluation task used by Chijiiwa and colleagues [25]. In this task, dogs first watched as a novel experimenter acted either prosocially, antisocially, or neutrally (i.e., did not interact) toward their owner. Then, dogs were released and could choose to take a high-value food reward from that actor or from a second neutral experimenter. Based on Chijiiwa and colleagues’ findings [25], we hypothesized that dogs would exhibit a negativity bias after witnessing an antisocial interaction with their owner. In contrast with Chijiiwa et al. [25], however, we also hypothesized that dogs could exhibit a positivity bias after witnessing a prosocial interaction with their owner. We made this prediction because, although the literature on social evaluation in dogs provides mixed results, at least some studies, e.g., [16,17,19,20,22], have provided evidence of a positivity bias in dogs in certain contexts and under certain conditions. Note that we specifically chose to use a social evaluation method that involved dogs’ owners since we hypothesized that attachment would be most likely to affect the dog’s performance when the recipient of the observed interaction was the individual most connected to the dogs.

After assessing the dogs’ performance on this task, we then assessed each dog’s attachment bonds to its owner and tested whether this predicted the dogs’ behavioral performance. Previous research has typically used one of two different methods to assess attachment relationships between dogs and their owners: behavioral assessments and owner-survey methods. Behavioral tests, such as the classic Strange Situation Test originally developed for research in human infants [53], typically use observational data to classify dogs’ individual attachment styles based on the changes in dogs’ behavior when their owner is present versus when their owner is absent. These behavioral tests, however, have several limitations. First, these tests often place dogs in intentionally stressful situations (e.g., by separating dogs from their owners when in an unfamiliar location, as in the Strange Situation Test or the Secure-Base Test, e.g., [45,47], or perhaps by placing dogs in the presence of a threatening individual, as in the Threatening Stranger Procedure [45,51]) which sometimes causes ethical concerns with dog owners. Secondly, these behavioral tests are often relatively long in duration, with the Strange Situation Test, for example, taking over twenty minutes to complete. Given the stress-inducing nature of many behavioral tests, in addition to the fact that the tests at our center are often shorter in duration, we worried that a long behavioral test like the Strange Situation Test could increase dogs’ frustration and anxiety during their visits. As a result, we instead opted to assess dogs’ attachment using an owner-survey method.

While various scales have been developed to assess owners’ attachment to their dogs—such as the Dog Attachment Questionnaire [54], the Lexington Attachment to Pets Scale [55], or the Monash Dog Owner Relationship Scale [56]—fewer owner-survey methods have been developed to assess dogs’ attachment to their owners. One questionnaire-based behavioral evaluation tool known as the Canine Behavioral Assessment & Research Questionnaire (C-BARQ), however, is widely used to explore a variety of canine personality traits, including attachment [57]. This standardized, 100-item validated assessment includes six internally consistent (α = 0.74) questions that explore the degree to which a dog displays attachment and attention-seeking behaviors toward their human owner (see Table 1) [57]. Notably, the attachment and attention-seeking behaviors assessed in the C-BARQ closely resemble the types of behavior examined during standard behavioral canine attachment assessments. For instance, both the C-BARQ and behavioral tests such as the Strange Situation Test or Secure-Base test examine the degree and invasiveness of proximity-seeking behavior that a dog demonstrates toward their owner as well as whether dogs display a preference for their attachment figure [46,47,57]. Additionally, the C-BARQ examines dogs’ reactions to interactions between the dog’s owner and an unfamiliar person, as in the Strange Situation Test [45,46] or the Threatening Stranger Procedure [45,51]. Thus, the attachment and attention-seeking subset of the C-BARQ appears to be a promising owner-based survey method for investigating the nature of dogs’ relationships with their owners that could be an optimal alternative when behavioral assessments are not feasible. As a result, we opted to use the attachment and attention-seeking subset of the C-BARQ to investigate whether attachment might impact dogs’ performance on an owner-based social evaluation task.

## 2. Part A: Do Dogs Socially Evaluate Individuals Who Interact with Their Owners

### 2.1. Methods

#### 2.1.1. Participants

Thirty-seven domesticated pet dogs (17 female, *M_age_* = 5.68 years, *SD_age_* = 2.82, range_age_ = 1–13) were tested alongside their owners (36 unique owners, 1 owner tested two pet dogs of the same household) at the Canine Cognition Center at Yale University (see Table S1 for additional demographic information). An additional 4 dogs (2 female) were excluded due to either owner (*N* = 2) or experimenter (*N* = 2) errors during the demonstration. Note that we had initially planned to test a full sample of 60 dogs, but due to a 19-month lapse in testing during the COVID-19 pandemic, we could test only 12–13 dogs in each of the conditions rather than the 20 that we had originally pre-registered. To underscore the reliability of our existing data, however, it was important to note that our final sample size was comparable to the original sample size in Chijiiwa et al. [25].

#### 2.1.2. Procedure

**Figure 1:**
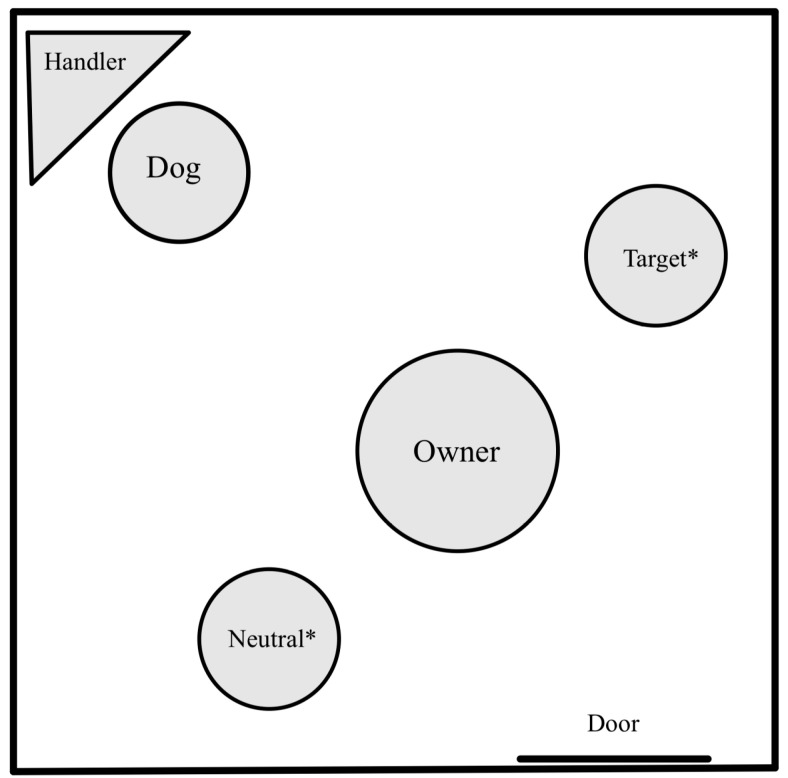
The test set up for our social evaluation study. The identities of the two actors (denoted by *) were counterbalanced between dogs and their positions were counterbalanced across trials.

We began by testing dogs in a social evaluation study closely modeled after the one used by Chijiiwa and colleagues [25] (see Figure 1 for the set-up). Owners sat in the middle of a testing room with two female experimenters (hereafter the *actors*), one on either side. One of the actors (hereafter the *target actor*, see three conditions below) interacted with the owner during the presentation, whereas the other remained neutral. The owner and the actors faced the dog subject, who was located in the corner of the testing room and handled by a third experimenter. Owners held a closed transparent Tupperware container with a red block (the target object) inside and were cued by the third experimenter to begin the demonstration. The demonstration consisted of the owner unsuccessfully attempting to open the lid of the container for approximately 10 s while the actors focused their gaze on the ground in front of them *(initial attempt period*). After the initial attempt period, the dogs then saw one of three different test conditions. In the *prosocial condition* (*N* = 13), the owner turned to present the container to one of the actors (the prosocial target actor) while the other actor remained neutral. The prosocial actor would then hold the base of the container so that the owner could successfully open the container. The owner then turned back to the dog, removed and held out the red block, then secured it back in the container. For the *antisocial condition* (*N* = 12), after the initial attempt period, the owner similarly turned to one of the actors (the antisocial target actor) while the other actor remained neutral. Rather than helping the owner, the antisocial actor looked at and made eye contact with the owner, then behaved uncooperatively by turning their entire body away from the owner for approximately 2 s. After this, the owner made 3 more seconds of failed attempts to open the container. In the *control condition* (*N* = 12), the owner did not turn to either actor to recruit help but instead paused for 2 s, looking straight at the container held out in front of them, while one actor (the control target actor) turned away. The owner then made a failed attempt for 3 more seconds to open the container. This allowed us to control whether the movement of the target actor alone affected the dogs’ choices. In all three conditions, the owner placed the container on the ground at the end of the demonstration.

The dogs were then presented with a choice between the two actors. During this *choice phase*, the target and neutral actors, still looking down, each extended their hands with a piece of high-value food (i.e., freeze-dried beef liver or, for dogs with food sensitivities, a comparable owner-provided treat), and the dog was released. The dog was allowed to receive only 1 reward, and our primary dependent measure was which actor the dogs approached first to receive their reward.

All dogs completed four trials of the same condition. Of the full sample, four trials were excluded due to either an owner error (*N* = 3) or the failure of the dog to approach an experimenter within 30 s of the trial’s onset (*N* = 1). The actors’ roles were counterbalanced between the dogs, and their positions were counterbalanced across the trials. Our secondary dependent measures were the total duration that the dogs spent looking at each of the actors during all four of the demonstrations as well as their average latency to choose either the target or neutral actor.

### 2.2. Results

**Figure 2:**
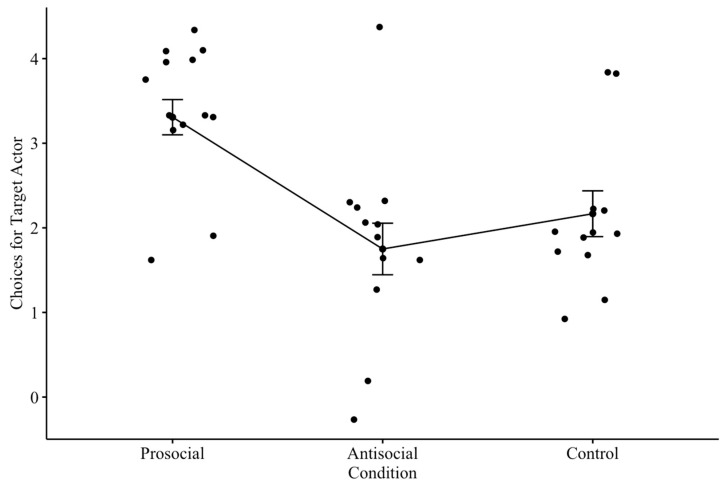
Jitter plot of dogs’ mean choices for target actor by condition. Notably, dogs chose the target actor at above chance levels in the prosocial condition but not in the antisocial and control conditions.

A Kruskal–Wallis test revealed the strong effect (ε^2^ = 0.41) of the condition on dogs’ target actor choices (*H*(2) = 14.58, *p* < 0.001) (see Figure 2). Pairwise Mann–Whitney U tests between the conditions (with Bonferroni adjusted *p*-value of 0.05/3 = 0.017) revealed that there were statistically significant differences between the control and prosocial conditions (*W* = 28, *p* = 0.004), as well as between the antisocial and prosocial conditions (*W* = 18, *p* < 0.001). There was no significant difference between the antisocial and control conditions (*W* = 84, *p* = 0.428). Additional pairwise Wilcoxon Signed-Rank tests were performed to determine if dogs preferred the target actor, as opposed to the neutral actor, in each condition (with Bonferroni adjusted *p* = 0.017). We found that dogs only had a statistically significant preference for the target actor in the prosocial condition (*V* = 66, *p* = 0.003). There were no statistically significant preferences toward the actor in the antisocial (*V* = 3.5, *p* = 0.713) nor the control conditions (*V* = 8, *p* = 0.357).

**Figure 3:**
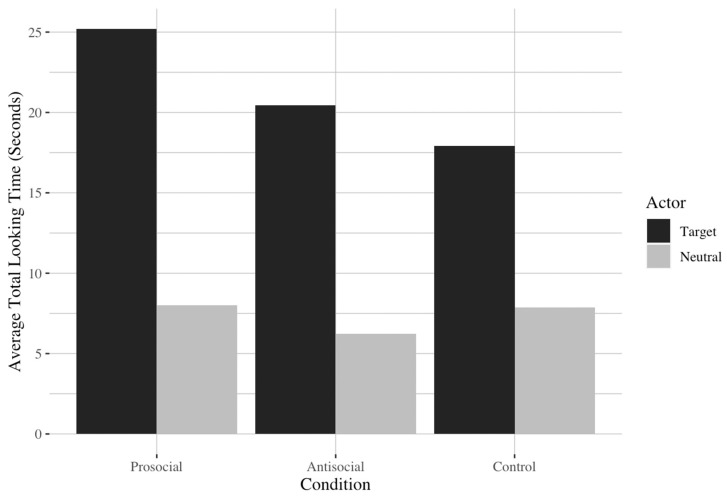
Dogs’ average looking time in seconds by actor and condition. Notably, dogs looked significantly longer at the target actor than the neutral actor in all three conditions.

Additional pairwise Wilcoxon Signed-Rank tests were performed to determine if the dogs looked longer at the target actor than the neutral actor during the demonstrations (Bonferroni adjusted *p* = 0.017) (see Table S2 for descriptive statistics). We found in all three conditions that the dogs looked significantly longer at the target actor than they did at the neutral actor (prosocial: *V* = 88, *p* = 0.001; antisocial: *V* = 76, *p* = 0.001; control: *V* = 74, *p* = 0.003), suggesting that dogs successfully visually distinguished between the two female actors in each condition (see Figure 3).

Lastly, we examined whether condition impacted dogs’ average latency per actor choice. The results of pairwise Wilcoxon Signed-Rank tests (with Bonferroni adjusted *p* = 0.017) revealed that dogs in the prosocial condition were significantly faster to choose to accept a food reward from the neutral actor than the prosocial actor (*V* = 74, *p* = 0.007), but no significant effect was found within antisocial (*V* = 29, *p* = 0.470) nor control (*V* = 47, *p* = 0.569) conditions (see Table S3 for descriptive statistics, see also Figure S1). Given that dogs significantly preferred the target actor in the prosocial condition, this finding that dogs were faster to make a choice for the neutral actor suggests that the dogs might have been thinking more critically about their choice when choosing the prosocial actor, therefore taking longer, and perhaps choosing quickly on impulse when choosing the neutral actor.

### 2.3. Discussion

Dogs successfully distinguished between the target and neutral actors across all three conditions, and dogs in the prosocial condition exhibited a significant preference for the prosocial actor compared to the neutral actor, while dogs in the antisocial and control conditions exhibited no preference. These results suggest that dogs prefer to interact with individuals who help their owners but show no clear evaluation of individuals who fail to help their owners. Note that our results differ from the previous findings observed by Chijiiwa et al. [25] in two ways. First, our results revealed a positivity bias that was not found in Chijiiwa et al. [25]—dogs in our study showed a significant bias toward the prosocial actor, whereas no such preference was observed by Chijiiwa and colleagues [25]. Second, we failed to observe the negativity bias pattern that Chijiiwa and colleagues [25] observed; in contrast to their results, our dog participants showed no avoidance of the antisocial actor relative to the neutral actor. The lack of a negativity bias found in our study is somewhat surprising, particularly given that research has shown that a negativity bias is privileged in human infant development, with infants as young as 3 months old avoiding antisocial experimenters, e.g., [4]. However, our results are consistent with other studies that show dogs as sometimes indicating a positivity bias for helpful experimenters, e.g., [15].

Given the variance in dogs’ evaluation performance in our study, the second part of our study then went on to test whether dogs’ attachment to their owners could explain the variation observed in their social evaluation performance. Historically, researchers have used two different methods to assess dogs’ attachment to their owners: measuring their behavioral performance, e.g., the classic Strange Situation Test [45], and owner survey methods, e.g., the C-BARQ [57]. We chose to use the C-BARQ survey method rather than a Strange Situation Test for a few reasons. First, the Strange Situation Test sometimes causes concerns with dog owners because it does cause some stress during the period when dogs are isolated. Second, the Strange Situation Test takes over twenty minutes to complete; since many of the tests we run at our center are shorter in duration, we worried that a long test like the Strange Situation Test would increase dogs’ frustration and anxiety during their visits. For these reasons, we chose to assess dogs’ attachment using the attachment questions developed in the C-BARQ instead.

We hypothesized that dogs who were shown by the C-BARQ to display more attachment and attention-seeking behaviors toward their owners (i.e., with stronger attachment bonds) would be more likely to prefer an actor who helped their owner than a neutral experimenter compared to dogs who displayed fewer attachment and attention-seeking behaviors (i.e., had weaker attachment bonds) to their owner. Additionally, we predicted that dogs with stronger attachment bonds to their owner would be more likely to prefer a neutral experimenter than an actor who did not help their owners compared to dogs who had weaker attachment bonds to their owners. Finally, we predicted that attachment would not affect dogs’ choices in the control condition. Taken together, we hypothesized that dogs with stronger attachment bonds to their owner would exhibit both stronger positivity and negativity biases compared to dogs with weaker attachment bonds to their owners.

## 3. Part B: Attachment as a Predictor of Actor Choices during Social Evaluation

### 3.1. Methods

#### 3.1.1. Participants

Twenty-six of the domesticated pet dogs from Part A (11 female, *M_age_* = 5.87 years, *SD_age_* = 2.74, range_age_ 1–13) were assessed by their owners for analysis in Part B after previously participating in Part A (see Table S1 for additional demographic information). Eleven dogs were excluded from the original thirty-seven due to the owner’s failure to complete the survey.

#### 3.1.2. Procedure

All owners who had participated in Part A were later administered a questionnaire-based assessment for research on domestic dogs called the C-BARQ [57]. The C-BARQ contains a variety of questions on dog behavior and temperament, including a section on attachment and attention-seeking behaviors specifically. We chose to use an average score on a scale from 0 (never) to 4 (always) from Questions 68–73 (see Table 1) of the C-BARQ [57] to measure the strength of dogs’ attachment bond to their owner. Thus, each dog received a numeric score from 0 to 4, detailing the strength of their attachment bond to their owner. Dogs who displayed more attachment and attention-seeking behaviors (i.e., displayed evidence of stronger attachment bonds) received higher scores.

### 3.2. Results

**Figure 4:**
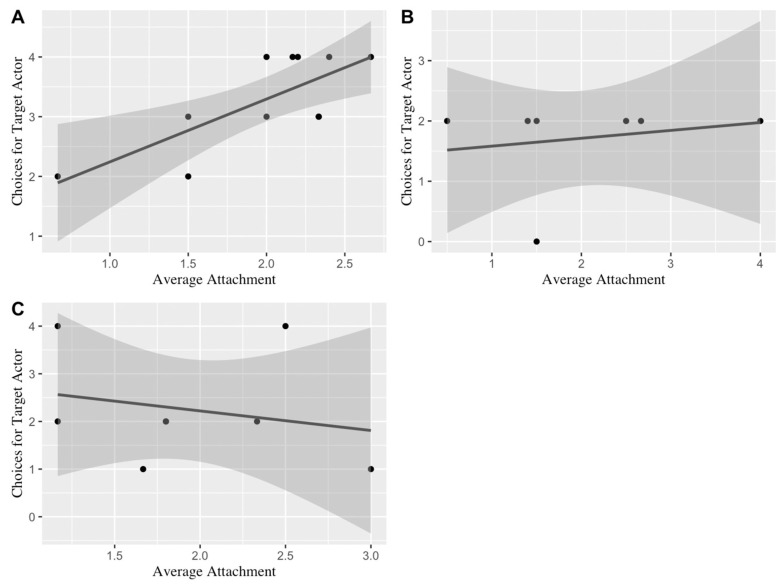
The relationship between the strength of dogs’ attachment bonds to their owners and their choices for the target actor in the prosocial (**A**), antisocial (**B**), and control conditions (**C**).

Simple linear regression was used to evaluate whether average attachment scores predicted the number of actor choices in each condition of Part A (using Bonferroni adjusted *p* = 0.017). In the prosocial model (*N* = 11), the results of the linear regression indicated that average attachment was a significant predictor of actor choices (*F*(1, 9) = 11.86, *p* = 0.007, *R*^2^*_adjusted_* = 0.52). The results of a Pearson correlation verified this result, as there was a strong significant positive association between the average attachment and choices for the target actor in the prosocial condition (*r*(9) = 0.75, *p* = 0.007) (see Figure 4A).

The results of the antisocial model (*N* = 7), however, revealed no significant prediction of attachment on actor choices (*F*(1,5) = 0.20, *p* = 0.672, *R*^2^*_adjusted_* = −0.15) (see Figure 4B). Additionally, a Pearson correlation test verified that there was no association between average attachment and actor choices in the antisocial condition (*r*(5) = 0.20, *p* = 0.672). Similarly, there was no significant prediction of attachment on actor choices in the control condition (*N* = 8, *F*(1,6) = 0.33, *p* = 0.589, *R*^2^*_adjusted_* = −0.11), and this result was confirmed by a Pearson correlation test (*r*(6) = −0.23, *p* = 0.589) (see Figure 4C). In other words, as the strength of attachment increased, dogs became more likely to prefer a prosocial actor compared to a neutral actor. On the other hand, the strength of attachment did not appear to have any relationship with the dogs’ preferences toward an antisocial actor or a control actor.

Additionally, simple linear regression was used to determine whether average attachment scores predicted how long dogs spent looking at the target actor within each condition (using Bonferroni adjusted *p* = 0.017). The results of the prosocial, antisocial, and control models revealed that attachment scores did not significantly predict how long dogs spent looking at the target actor (prosocial: *F*(1,9) = 1.18, *p* = 0.305, *R*^2^*_adjusted_* = 0.02; antisocial: *F*(1,5) = 0.10, *p* = 0.768, *R*^2^*_adjusted_* = −0.18; control: *F*(1,6) = 0.06, *p* = 0.817, *R*^2^*_adjusted_* = −0.16). These results were also confirmed using Pearson correlation tests (prosocial: *r*(9) = 0.34, *p* = 0.305; antisocial: *r*(5) = 0.14, *p* = 0.768; control: *r*(6) = −0.10, *p* = 0.817).

Lastly, simple linear regression was used to determine whether average attachment scores predicted how quickly the dogs chose to accept a reward from the target actor within each condition (using Bonferroni adjusted *p* = 0.017). The average attachment was not a significant predictor of dogs’ average choice latency for the target actor in any of the three conditions (prosocial: *F*(1,9) = 0.004, *p* = 0.951, *R*^2^*_adjusted_* = −0.11; antisocial: *F*(1,5) = 0.20, *p* = 0.671, *R*^2^*_adjusted_* = −0.15; control: *F*(1,6) = 3.53, *p* = 0.109, *R*^2^*_adjusted_* = 0.27). These results were confirmed using Pearson correlation tests (prosocial: *r*(9) = −0.02, *p* = 0.951; antisocial: *r*(5) = 0.20, *p* = 0.671; control: *r*(6) = −0.61, *p* = 0.109).

### 3.3. Discussion

Dogs’ average attachment was a significant predictor of preference toward prosocial actors in an owner-centered social evaluation paradigm. Specifically, dogs with stronger attachment bonds were significantly more likely to prefer an actor who helped their owner compared to dogs with weaker attachment bonds. Interestingly, dogs with stronger attachment bonds did not have a stronger aversion to the antisocial actor. Indeed, attachment did not seem to have any effect on whether dogs chose to approach the antisocial or neutral actor in the antisocial condition in contrast with our predictions. Our findings suggest that dogs who have stronger attachment bonds to their owners are more likely to positively socially evaluate people who help their owners but do not seem to evaluate and avoid people who refuse to help their owners.

## 4. General Discussion

The goal of the present study was to determine whether we could explain dogs’ mixed performance in social evaluation studies with a factor known to affect how individuals relate to one another: attachment bonds. To examine this question, we first replicated a previous test to determine dogs’ social evaluation [25] and then tested whether the dogs’ performance was mediated by the strength of their attachment bonds to their owner as measured by the attachment and attention-seeking subset of an owner-administered C-BARQ survey. In Part A of our study, we found that dogs exhibited a positivity bias but not a negativity bias in their social evaluation. Dogs significantly preferred to interact with people who helped their owners, but showed no avoidance of individuals who actively refused to help their owners. In Part B, we found evidence that dogs’ attachment bonds to their owner significantly predicted their performance in the prosocial condition but did not predict their choices in the antisocial and control conditions. Taken together, our findings provide early evidence that attachment may predict meaningful individual differences across dogs during social evaluation tasks.

Our canine findings align nicely with previous results in humans, which have demonstrated that attachment impacts how people behave in [29,30,31,32] and physiologically respond to [31] a variety of social situations. Given the evidence that a similar attachment system may be present in dogs, e.g., [38,39,40,41,42,43,44,45,46,47,48,49,51,52], it makes sense that we observed attachment playing a comparable role in dogs’ evaluations of actors who help their owners.

Our findings not only provide support that attachment affects dogs’ performance in social evaluation studies but also may shed some light on the mixed results that have been observed to date within existing social evaluation research. Researchers have long observed inconsistent patterns in dogs’ performance on canine social evaluation tasks, with some studies finding that dogs successfully evaluated prosocial and antisocial actors [15,16,17,18,25] and others finding no evidence for successful evaluation [19,20,21,22,23,24]. Our results provide a hint on why researchers may have observed such varying patterns of performance. Specifically, since the nature of a dog’s relationship to their owner can vary substantially, not just between individual dogs but also between populations of dogs, it is possible that studies showing stronger social evaluation effects may have happened to test dogs who have stronger attachment bonds to their owners.

Indeed, there are hints in the existing literature that attachment may play more of a role in dogs’ mixed performance than previous works have recognized. Consider, for example, the mixed results of Silver et al. [15], which found that trained agility dogs exhibited a strong positivity bias during social evaluation, but untrained pet dogs did not. Silver et al. [15] initially argued that training might impact dogs’ capacity for social evaluation. Our results provide a new take on this interpretation—suggesting that highly trained dogs may develop stronger attachment bonds compared to untrained dogs. This hypothesis is supported by the research of Fallani and colleagues [38], showing that highly trained guide dogs tend to have stronger attachment bonds to their owner than untrained pet dogs. In this way, future research should consider investigating the relationship between attachment and training in dogs and the role that both of these factors play in dogs’ social evaluation performance.

Interestingly, our findings in Part A stand in contrast to the results of Chijiiwa and their colleagues’ study [25], which used the same social evaluation paradigm that we used in our study. Chijiiwa and colleagues [25] observed that dogs showed a negativity bias in their task but no positivity bias; however, we found an opposite pattern of performance. Although we hypothesized in Part B that dogs who displayed more attachment and attention-seeking behaviors (i.e., had stronger attachment bonds) would result in a stronger aversion to antisocial behavior toward their owner, it is possible that we did not observe an effect of attachment in this condition since we did not see any behavioral evidence of a negativity bias in Part A. Future work should attempt to alter the antisocial condition to make the act of avoidance more salient. For example, work in human infants has shown that participants often show a stronger avoidance of antisocial actors in scenarios in which an antisocial actor takes an object or actively hinders another individual [58]—it is possible that we would observe more robust social evaluations in the antisocial condition if we used a more salient antisocial action.

One limitation of our design concerns our use of the C-BARQ to operationalize attachment. Given that we found a significant effect of attachment, it can be reasonably assumed that our method had sound construct validity. It is important to note, however, that the previous literature on attachment in dogs has primarily measured this construct using behavioral assessments such as the Strange Situation Test. As a result, future research should aim to employ this test. A second limitation of the current design is that we may not have seen any effect of attachment on an antisocial scenario because we did not isolate specific attachment styles. The C-BARQ allowed us to assess dogs who displayed fewer attachment and attention-seeking behaviors and who we interpreted to have weaker attachment bonds, which appeared behaviorally similar to dogs with avoidant attachment styles. However, more attachment and attention-seeking behaviors could possibly be an indicator of either secure or anxious attachment; however, these attachment styles typically result in very different behavior. For instance, work in humans has shown that anxious children are far less likely to explore a novel environment than secure children [53]. Future work could thus profit from using more fine-grained measures of dog attachment to test the role that this individual difference plays in social evaluation.

## 5. Conclusions

The present results suggest that, just as in humans, a dog’s pattern of attachment may be an important individual difference that affects their social evaluation skills and behavior. We found that dogs not only tended to prefer actors that helped their owner over neutral actors but also that dogs who displayed more attachment and attention-seeking behaviors (i.e., appear to have stronger attachment bonds) toward their owners were significantly more likely to exhibit this positivity bias. These results provide promising evidence that attachment may be a meaningful variable to analyze in future canine social evaluation research and open avenues for new work on the relationship between attachment and social cognition in non-human species more broadly.

## Figures and Tables

**Table 1:** C-BARQ attachment and attention-seeking questions, see [57].

Question Number	Thinking Back over the Recent Past, How Often Has Your Dog Shown the Following Signs of Attachment or Attention-Seeking on a Scale from 0 (Never) to 4 (Always):
68	Displays a strong attachment for one particular member of the household.
69	Tends to follow you (or other members of the household) about the house, from room to room.
70	Tends to sit close to, or in contact with, you (or others) when you are sitting down.
71	Tends to nudge, nuzzle, or paw you (or others) for attention when you are sitting down.
72	Becomes agitated (whines, jumps up, tries to intervene) when you (or others) show affection for another person.
73	Becomes agitated (whines, jumps up, tries to intervene) when you (or others) show affection for another dog or animal.

## Data Availability

The data presented in this study are available upon reasonable request from the corresponding author. The data are not publicly available due to privacy and ethical reasons.

## Supplementary Materials

The following supporting information can be downloaded at: https://www.mdpi.com/article/10.3390/ani13152480/s1, Table S1: Demographic information for Part A (participants 1–37) and Part B (participants 1–26), Table S2: Descriptive statistics for average total looking time (seconds) per actor per condition, Table S3: Descriptive statistics for average response latency (seconds) per actor per condition, Figure S1: Dogs’ average response latency in seconds by actor and condition.

## References

[1] Abdai J., Miklósi Á. (2016). The Origin of Social Evaluation, Social Eavesdropping, Reputation Formation, Image Scoring or What You Will. Front. Psychol..

[2] Melis A.P., Hare B., Tomasello M. (2006). Chimpanzees Recruit the Best Collaborators. Science.

[3] Hamlin J.K., Wynn K., Bloom P. (2007). Social Evaluation by Preverbal Infants. Nature.

[4] Hamlin J.K., Wynn K., Bloom P. (2010). Three-Month-Olds Show a Negativity Bias in Their Social Evaluations. Dev. Sci..

[5] Hamlin J.K., Wynn K. (2011). Young Infants Prefer Prosocial to Antisocial Others. Cogn. Dev..

[6] Hamlin J.K. (2015). The Case for Social Evaluation in Preverbal Infants: Gazing toward One’s Goal Drives Infants’ Preferences for Helpers over Hinderers in the Hill Paradigm. Front. Psychol..

[7] Hamlin J.K. (2014). Context-Dependent Social Evaluation in 4.5-Month-Old Human Infants: The Role of Domain-General versus Domain-Specific Processes in the Development of Social Evaluation. Front. Psychol..

[8] Krupenye C., Hare B. (2018). Bonobos Prefer Individuals That Hinder Others over Those That Help. Curr. Biol..

[9] Kawai N., Yasue M., Banno T., Ichinohe N. (2014). Marmoset Monkeys Evaluate Third-Party Reciprocity. Biol. Lett..

[10] Anderson J.R., Kuroshima H., Takimoto A., Fujita K. (2013). Third-Party Social Evaluation of Humans by Monkeys. Nat. Commun..

[11] Anderson J.R., Takimoto A., Kuroshima H., Fujita K. (2013). Capuchin Monkeys Judge Third-Party Reciprocity. Cognition.

[12] Subiaul F., Vonk J., Okamoto-Barth S., Barth J. (2008). Do Chimpanzees Learn Reputation by Observation? Evidence from Direct and Indirect Experience with Generous and Selfish Strangers. Anim. Cogn..

[13] Herrmann E., Keupp S., Hare B., Vaish A., Tomasello M. (2013). Direct and Indirect Reputation Formation in Nonhuman Great Apes (Pan Paniscus, Pan Troglodytes, Gorilla Gorilla, Pongo Pygmaeus) and Human Children (Homo Sapiens). J. Comp. Psychol..

[14] Russell Y.I., Call J., Dunbar R.I.M. (2008). Image Scoring in Great Apes. Behav. Process..

[15] Silver Z.A., Furlong E.E., Johnston A.M., Santos L.R. (2021). Training Differences Predict Dogs’ (Canis Lupus Familiaris) Preferences for Prosocial Others. Anim. Cogn..

[16] Kundey S.M.A., De Los Reyes A., Royer E., Molina S., Monnier B., German R., Coshun A. (2011). Reputation-like Inference in Domestic Dogs (Canis Familiaris). Anim. Cogn..

[17] Carballo F., Freidin E., Putrino N., Shimabukuro C., Casanave E., Bentosela M. (2015). Dog’s Discrimination of Human Selfish and Generous Attitudes: The Role of Individual Recognition, Experience, and Experimenters’ Gender. PLoS ONE.

[18] Anderson J.R., Bucher B., Chijiiwa H., Kuroshima H., Takimoto A., Fujita K. (2017). Third-Party Social Evaluations of Humans by Monkeys and Dogs. Neurosci. Biobehav. Rev..

[19] Marshall-Pescini S., Passalacqua C., Ferrario A., Valsecchi P., Prato-Previde E. (2011). Social Eavesdropping in the Domestic Dog. Anim. Behav..

[20] Nitzschner M., Kaminski J., Melis A., Tomasello M. (2014). Side Matters: Potential Mechanisms Underlying Dogs’ Performance in a Social Eavesdropping Paradigm. Anim. Behav..

[21] Nitzschner M., Melis A.P., Kaminski J., Tomasello M. (2012). Dogs (Canis Familiaris) Evaluate Humans on the Basis of Direct Experiences Only. PLoS ONE.

[22] Freidin E., Putrino N., D’Orazio M., Bentosela M. (2013). Dogs’ Eavesdropping from People’s Reactions in Third Party Interactions. PLoS ONE.

[23] McAuliffe K., Bogese M., Chang L.W., Andrews C.E., Mayer T., Faranda A., Hamlin J.K., Santos L.R. (2019). Do Dogs Prefer Helpers in an Infant-Based Social Evaluation Task?. Front. Psychol..

[24] Jim H.-L., Plohovich M., Marshall-Pescini S., Range F. (2022). Wolves and Dogs Fail to Form Reputations of Humans after Indirect and Direct Experience in a Food-Giving Situation. PLoS ONE.

[25] Chijiiwa H., Kuroshima H., Hori Y., Anderson J.R., Fujita K. (2015). Dogs Avoid People Who Behave Negatively to Their Owner: Third-Party Affective Evaluation. Anim. Behav..

[26] Bowlby J. (1982). Attachment and Loss: Retrospect and Prospect. Am. J. Orthopsychiatry.

[27] Benoit D., Parker K.C.H. (1994). Stability and Transmission of Attachment across Three Generations. Child Dev..

[28] Waters E., Hamilton C.E., Weinfield N.S. (2000). The Stability of Attachment Security from Infancy to Adolescence and Early Adulthood: General Introduction. Child Dev..

[29] Mikulincer M. (1997). Adult Attachment Style and Information Processing: Individual Differences in Curiosity and Cognitive Closure. J. Pers. Soc. Psychol..

[30] Renner B. (2006). Curiosity About People: The Development of a Social Curiosity Measure in Adults. J. Pers. Assess..

[31] Vrtička P., Andersson F., Grandjean D., Sander D., Vuilleumier P. (2008). Individual Attachment Style Modulates Human Amygdala and Striatum Activation during Social Appraisal. PLoS ONE.

[32] Gillath O., Giesbrecht B., Shaver P.R. (2009). Attachment, Attention, and Cognitive Control: Attachment Style and Performance on General Attention Tasks. J. Exp. Soc. Psychol..

[33] Holvoet C., Scola C., Arciszewski T., Picard D. (2016). Infants’ Preference for Prosocial Behaviors: A Literature Review. Infant Behav. Dev..

[34] Johnson S.C., Dweck C.S., Chen F.S. (2007). Evidence for Infants’ Internal Working Models of Attachment. Psychol. Sci..

[35] Johnson S.C., Dweck C.S., Chen F.S., Stern H.L., Ok S.-J., Barth M. (2010). At the Intersection of Social and Cognitive Development: Internal Working Models of Attachment in Infancy. Cogn. Sci..

[36] Beck L., Madresh E.A. (2008). Romantic Partners and Four-Legged Friends: An Extension of Attachment Theory to Relationships with Pets. Anthrozoös.

[37] Kurdek L.A. (2009). Pet Dogs as Attachment Figures for Adult Owners. J. Fam. Psychol..

[38] Fallani G., Previde E.P., Valsecchi P. (2006). Do Disrupted Early Attachments Affect the Relationship between Guide Dogs and Blind Owners?. Appl. Anim. Behav. Sci..

[39] Fallani G., Prato Previde E., Valsecchi P. (2007). Behavioral and Physiological Responses of Guide Dogs to a Situation of Emotional Distress. Physiol. Behav..

[40] Gácsi M., Topál J., Miklósi Á., Dóka A., Csányi V. (2001). Attachment Behavior of Adult Dogs (Canis Familiaris) Living at Rescue Centers: Forming New Bonds. J. Comp. Psychol..

[41] Palestrini C., Previde E.P., Spiezio C., Verga M. (2005). Heart Rate and Behavioural Responses of Dogs in the Ainsworth’s Strange Situation: A Pilot Study. Appl. Anim. Behav. Sci..

[42] Parthasarathy V., Crowell-Davis S.L. (2006). Relationship between Attachment to Owners and Separation Anxiety in Pet Dogs (Canis Lupus Familiaris). J. Vet. Behav..

[43] Prato-Previde E., Custance D.M., Spiezio C., Sabatini F. (2003). Is the Dog-Human Relationship an Attachment Bond? An Observational Study Using Ainsworth’s Strange Situation. Behaviour.

[44] Rehn T., McGowan R.T.S., Keeling L.J. (2013). Evaluating the Strange Situation Procedure (SSP) to Assess the Bond between Dogs and Humans. PLoS ONE.

[45] Solomon J., Beetz A., Schöberl I., Gee N., Kotrschal K. (2019). Attachment Security in Companion Dogs: Adaptation of Ainsworth’s Strange Situation and Classification Procedures to Dogs and Their Human Caregivers. Attach. Hum. Dev..

[46] Topál J., Miklósi Á., Csányi V., Dóka A. (1998). Attachment Behavior in Dogs (Canis Familiaris): A New Application of Ainsworth’s (1969) Strange Situation Test. J. Comp. Psychol..

[47] Udell M.A.R., Brubaker L., Thielke L.E., Wanser S.S.H., Rosenlicht G., Vitale K.R., Anderson J.R., Kuroshima H. (2021). Dog–Human Attachment as an Aspect of Social Cognition: Evaluating the Secure Base Test. Comparative Cognition: Commonalities and Diversity.

[48] Brubaker L., Udell M.A.R. (2023). Does Pet Parenting Style Predict the Social and Problem-Solving Behavior of Pet Dogs (Canis Lupus Familiaris)?. Anim. Cogn..

[49] Valsecchi P., Previde E.P., Accorsi P.A., Fallani G. (2010). Development of the Attachment Bond in Guide Dogs. Appl. Anim. Behav. Sci..

[50] Horn L., Huber L., Range F. (2013). The Importance of the Secure Base Effect for Domestic Dogs—Evidence from a Manipulative Problem-Solving Task. PLoS ONE.

[51] Gácsi M., Maros K., Sernkvist S., Faragó T., Miklósi Á. (2013). Human Analogue Safe Haven Effect of the Owner: Behavioural and Heart Rate Response to Stressful Social Stimuli in Dogs. PLoS ONE.

[52] Topál J., Gácsi M., Miklósi Á., Virányi Z., Kubinyi E., Csányi V. (2005). Attachment to Humans: A Comparative Study on Hand-Reared Wolves and Differently Socialized Dog Puppies. Anim. Behav..

[53] Ainsworth M.D.S., Blehar M.C., Waters E., Wall S.N. (2015). Patterns of Attachment: A Psychological Study of the Strange Situation.

[54] Archer J., Ireland J.L. (2011). The Development and Factor Structure of a Questionnaire Measure of the Strength of Attachment to Pet Dogs. Anthrozoös.

[55] Johnson T.P., Garrity T.F., Stallones L. (1992). Psychometric Evaluation of the Lexington Attachment to Pets Scale (Laps). Anthrozoös.

[56] Dwyer F., Bennett P.C., Coleman G.J. (2006). Development of the Monash Dog Owner Relationship Scale (MDORS). Anthrozoös.

[57] Hsu Y., Serpell J. (2003). Development and Validation of a Questionnaire for Measuring Behavior and Temperament Traits in Pet Dogs. J. Am. Vet. Med. Assoc..

[58] Margoni F., Surian L. (2018). Infants’ Evaluation of Prosocial and Antisocial Agents: A Meta-Analysis. Dev. Psychol..

