# Supplementary material for: Impact of the Dog–Human Bond on Canine Social Evaluation: Attachment Predicts Preference toward Prosocial Actors
Source: Animals (Basel). 2023 Aug 1;13(15):2480. doi: 10.3390/ani13152480 (PMC10417759; doi:10.3390/ani13152480)
Supplement: Supplementary file 1 [file animals-13-02480-s001.zip › animals-2506201-supplementary.pdf]

Table S1. Demographic information for Part A (participants 1-37) and Part B (participants 1-26).

Participant	Condition	Breed	Age (Years)	Sex
1	Antisocial	Labrador Retriever / Springer Spaniel Mix	2.25	M
2	Prosocial	Golden Retriever	6.67	M
3	Control	Yorkshire Terrier	5.17	M
4	Prosocial	Terrier / Chihuahua / Australian Shepherd Mix	3.08	M
5	Prosocial	English Springer Spaniel	3.83	M
6	Prosocial	Labrador Retriever	8.08	M
7	Prosocial	Golden Retriever	10.67	F
8	Control	Pug	3.42	F
9	Antisocial	Golden Retriever	3.92	M
10	Prosocial	Terrier Mix	6.75	M
11	Prosocial	Poodle Mix	7.42	M
12	Control	Labrador Retriever	5.33	F
13	Control	Standard Poodle	7.33	F
14	Antisocial	Collie	8.42	M
15	Prosocial	Collie	4.08	M
16	Antisocial	Chesapeake Bay Retriever	10.67	F
17	Control	Vizsla / Shih-Tsu / Springer Spaniel Mix	6.08	M
18	Antisocial	Miniature Schnauzer	3.92	M
19	Antisocial	Rottweiler Mix	7.08	F
20	Control	Hound Mix	5.17	F
21	Prosocial	Norfolk Terrier Mix	5.50	F
22	Control	Chihuahua / Dachshund Mix	3.58	M
23	Prosocial	German Shepherd	3.67	M
24	Antisocial	Jack Russel Terrier / Basset Hound / Pembroke Welsh Corgi Mix	1.00	F
25	Prosocial	Labrador Retriever	12.58	F
26	Control	Labrador Retriever Mix	6.92	F
27	Control	Collie / Labrador Retriever / Irish Setter	9.08	F
28	Control	Miniature Schnauzer	3.50	F
29	Antisocial	Labrador Retriever Mix	5.75	M
30	Antisocial	Golden Retriever / Poodle Mix	4.92	M
31	Prosocial	German Shepherd / Labrador Retriever Mix	9.08	M
32	Prosocial	Pembroke Welsh Corgi	3.33	F
33	Antisocial	Chihuahua / Pomeranian / Shih-Tzu Mix	2.83	F
34	Control	Jack Russell Terrier Mix	10.92	M
35	Antisocial	Cavalier King Charles Spaniel	3.25	M
36	Control	Unknown Mix	2.92	F
37	Antisocial	Vizsla	1.92	F

Note: Breed was determined using owner-reports.

Table S2. Descriptive statistics for average total looking time (seconds) per actor per condition

Condition	Mean (target)	Standard Deviation (target)	Mean (neutral)	Standard Deviation (neutral)
Prosocial	25.20	10.58	8.02	9.48
Antisocial	20.44	11.75	6.23	3.94
Control	17.91	7.29	7.86	6.51

Table S3. Descriptive statistics for average response latency (seconds) per actor per condition

Condition	Mean (target)	Standard Deviation (target)	Mean (neutral)	Standard Deviation (neutral)
Prosocial	3.81	3.56	0.67	0.91
Antisocial	2.96	4.19	2.38	1.80
Control	3.17	3.24	2.04	1.92

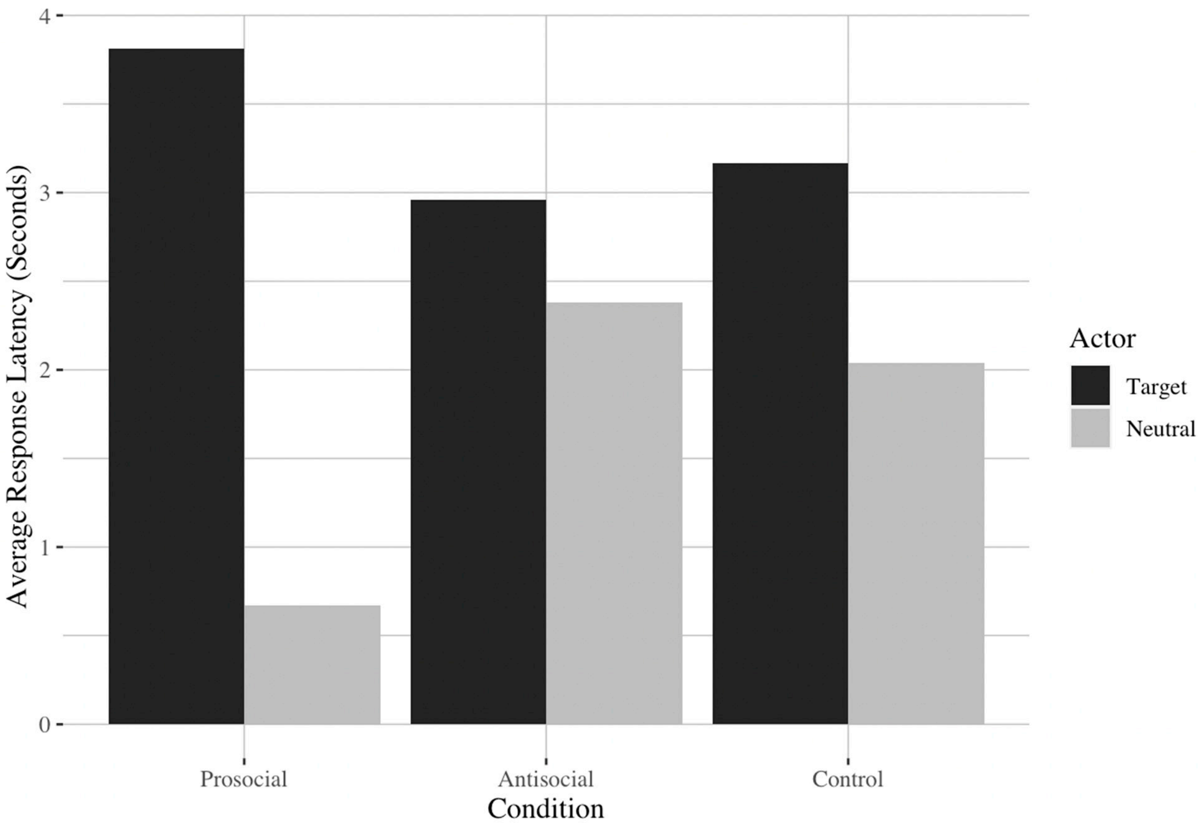

Figure S1. Dogs' average response latency in seconds by actor and condition. Notably, dogs were faster to choose to take a food reward from the neutral actor prosocial condition but not in antisocial or control conditions.
